# Effect of Melatonin on Tumor Growth and Angiogenesis in Xenograft Model of Breast Cancer

**DOI:** 10.1371/journal.pone.0085311

**Published:** 2014-01-09

**Authors:** Bruna Victorasso Jardim-Perassi, Ali S. Arbab, Lívia Carvalho Ferreira, Thaiz Ferraz Borin, Nadimpalli R. S. Varma, A. S. M. Iskander, Adarsh Shankar, Meser M. Ali, Debora Aparecida Pires de Campos Zuccari

**Affiliations:** 1 Department of Biology, Universidade Estadual Paulista, São José do Rio Preto, São Paulo, Brazil; 2 Laboratório de Investigação Molecular no Câncer, Department of Molecular Biology, Faculdade de Medicina de São José do Rio Preto, São José do Rio Preto, São Paulo, Brazil; 3 Cellular and Molecular Imaging Laboratory, Department of Radiology, Henry Ford Hospital, Detroit, Michigan, United States of America; 4 Department of Molecular Biology, Faculdade de Medicina de São José do Rio Preto, São José do Rio Preto, São Paulo, Brazil; University of Dundee, United Kingdom

## Abstract

As neovascularization is essential for tumor growth and metastasis, controlling angiogenesis is a promising tactic in limiting cancer progression. Melatonin has been studied for their inhibitory properties on angiogenesis in cancer. We performed an *in vivo* study to evaluate the effects of melatonin treatment on angiogenesis in breast cancer. Cell viability was measured by MTT assay after melatonin treatment in triple-negative breast cancer cells (MDA-MB-231). After, cells were implanted in athymic nude mice and treated with melatonin or vehicle daily, administered intraperitoneally 1 hour before turning the room light off. Volume of the tumors was measured weekly with a digital caliper and at the end of treatments animals underwent single photon emission computed tomography (SPECT) with Technetium-99m tagged vascular endothelial growth factor (VEGF) C to detect *in vivo* angiogenesis. In addition, expression of pro-angiogenic/growth factors in the tumor extracts was evaluated by membrane antibody array and collected tumor tissues were analyzed with histochemical staining. Melatonin *in vitro* treatment (1 mM) decreased cell viability (p<0.05). The breast cancer xenografts nude mice treated with melatonin showed reduced tumor size and cell proliferation (Ki-67) compared to control animals after 21 days of treatment (p<0.05). Expression of VEGF receptor 2 decreased significantly in the treated animals compared to that of control when determined by immunohistochemistry (p<0.05) but the changes were not significant on SPECT (p>0.05) images. In addition, there was a decrease of micro-vessel density (Von Willebrand Factor) in melatonin treated mice (p<0.05). However, semiquantitative densitometry analysis of membrane array indicated increased expression of epidermal growth factor receptor and insulin-like growth factor 1 in treated tumors compared to vehicle treated tumors (p<0.05). In conclusion, melatonin treatment showed effectiveness in reducing tumor growth and cell proliferation, as well as in the inhibition of angiogenesis.

## Introduction

Breast cancer is the most common type of cancer in women [1] with increasing incidence and mortality which is becoming a global public health problem [2], [3]. Furthermore, breast cancer has a high mortality rate, mainly due to tumor progression and metastatic spread, which require neovascularization [4]–[6].

Tumor growth has traditionally been associated with angiogenesis, which is the formation of new blood vessels from the pre-existing vasculature. Angiogenesis is controlled by pro-angiogenic and anti-angiogenic factors in the body. The angiogenesis is regulated by several growth factors such as vascular endothelial growth factor (VEGF), platelet-derived growth factor (PDGF), epidermal growth factor (EGF), Angiogenin, etc [7]. Recent studies have demonstrated that tumor progression can also occur through vasculogenesis. Vasculogenesis is typically followed by classical sprouting angiogenesis, where blood vessels are formed de novo by *in situ* differentiation of the primitive progenitors - i.e. angioblasts - into mature endothelial cells, which was thought to only take place during embryonic development [8], [9].

Once a tumor exceeds a few millimeters in diameter, hypoxia triggers a cascade of events to allow angiogenesis and tumor progression [8]. Hypoxia results in expression of VEGF, a potent endothelial cell mitogen [10]–[13]. VEGF is composed of a family of five isoforms denominated VEGF-A, VEGF-B, VEGF-C, VEGF-D and placental growth factor (PGF). Each of these factors can activate one or more receptors (VEGFR1, VEGFR2 and VEGFR3), promoting angiogenesis through its ability to stimulate the growth, migration and invasion of endothelial cells [14]–[17].

Hypoxia also up-regulates the expression of stromal-cell-derived factor-1α (SDF-1α), which can recruit pro-angiogenic cells from bone marrow [18], [19].Hypoxia can also act in the regulation of RANTES a chemokine of inflammatory cells [20], [21]. Other signalling pathways such as basic fibroblast growth factor (bFGF) and the receptor tyrosine kinase of angiopoietin-1 (Tie-2) can influence angiogenesis by increasing tumor invasion [22], [23], and vasculogenesis by mobilizing endothelial progenitor cells [9]. Given the variety of signals involved in the formation of new blood vessels, several proteins can be therapeutic targets. Therefore, it becomes extremely important to identify the most susceptible target to a particular treatment, and to develop effective therapies that involve a combination of several factors [8], [9].

Administration of melatonin, a hormone naturally produced and secreted in the pineal gland, appears to play an important role in tumor growth inhibition [24], [25] and different mechanisms of action have been proposed [26]. The action of melatonin is especially effective in estrogen receptor (ER)-positive breast cancer by reducing the mitogenic response of cells [27]. Still, melatonin can exert immunomodulatory and antiproliferative effects and act as antioxidants [28]–[32]. Furthermore, it has demonstrated that the pharmacological concentration of melatonin can inhibit angiogenesis directly or indirectly [33]–[35]. Recently, some studies have shown that melatonin can decrease the expression of Hypoxia Inducible Factor 1 alpha (HIF-1α) and VEGF in various cancers [5], [36]–[37].

The purposes of this study were to determine: 1) whether melatonin therapy effectively would decrease the size of implanted human triple negative breast cancer in a mouse model, 2) whether there would be changes in the expression of VEGF receptors determined by *in vivo* SPECT scanning in mice treated with melatonin or vehicle, 3) the changes in expression of angiogenic factors in tumors in response to melatonin. The ultimate goal was achieved by determining the therapeutic effectiveness of melatonin.

## Materials and Methods

### Ethics statement

All animal experiments were performed according to National Institutes of Health guideline and the protocol was approved by Institutional Animal Care and User Committee (IACUC No. 1203) of Henry Ford Health System.

### In vitro study

#### Cell culture

Cell culture. Triple negative human breast cancer cells (MDA-MB-231) (ATCC, Manassas, VA, USA) were cultured in 75 cm^2^ culture flasks with Dulbecco's modified Eagle's medium (DMEM) (GIBCO, Grand Island, NY, USA) supplemented with 10% fetal bovine serum (GIBCO, Grand Island, NY, USA), penicillin (100 IU/mL) and streptomycin (100 µg/mL) (GIBCO, Grand Island, NY, USA)until they were 80–90% confluent.

#### Cell viability by MTT (3-(4,5-Dimethylthiazol-2-yl)-2,5-diphenyltetrazolium bromide) assay

Individual well of a 96-well plate was inoculated with 100 µl of medium containing 5×10^4^ cells. Cells were incubated in medium with different concentrations of melatonin (0.0001 mM, 0.001 mM, 0.01 mM, 0.1 mM and 1 mM) for 24 h. Melatonin was diluted in ethanol 0.05%. In control cells, equivalent amount of ethanol was added as vehicle. Thereafter, 10 µL of MTT solution (ATCC, Manassas, VA, USA) were added to each well and the plates were incubated at 37°C for an additional 4 h. To solubilize the MTT formazan crystals, the cells were incubated with detergent (ATCC, Manassas, VA, USA) overnight at 37°C. Absorbance was measured at 570 nm by ELISA Plate reader (VICTOR3 – PerkinElmer, Waltham, MA, USA). Medium was used as background and subtracted from the samples. Cell viability (%) was calculated for all groups compared to control sample. All experimental samples were in triplicate.

### Animal model

Female athymic mice, 7–8 weeks of age and 25 g in weight (Charles River Laboratory, Inc.) were housed. MDA-MB-231, cultured *in vitro*, was harvested and re-suspended serum free media at a concentration of 6×10^7^ cells/ml. The animals received subcutaneous injection of 50 µl of cell suspension (3 million cells) in the right mammary gland or hind flank. These tumor cells are efficient in making xenografted tumors with almost 90% efficiency and show marked vascularity with less central necrosis. Two different implantation sites were chosen to determine whether treatment effects will differ based on implantation sites.

### Melatonin administration

Animals were randomly assigned to either the melatonin administration (n = 5) or the control group (vehicle treated, n = 8). Vehicle solution was prepared with 8 ml of phosphate buffered saline (PBS), 1 ml of dimethyl sulfoxide (DMSO) and 1 ml of Cremophor (Sigma, St. Louise, MO, USA). The animals of the control group received 100 µl of vehicle solution by intraperitoneal injection (IP).

Melatonin (Sigma, St. Louise, MO, USA) was diluted in vehicle and the animals from the melatonin group received IP of 100 µl of melatonin treatment (at dose of 40 mg/kg of body weight) for five days a week. Melatonin was administered 1 hr before room lighting was switched off. Administration of melatonin prior to the nocturnal increase in endogenous melatonin may be most effective because tissues are most sensitive to the hormone at this time [38], [39].

Melatonin or vehicle administration started on the day of tumor implantation (soon after implantation) and continued for five days a week for 21 days. On the 22nd day, all animals underwent SPECT scanning with Tc-99m-HYNIC-VEGF-c followed by euthanasia and collection of tumors for immunohistochemistry and membrane antibody array to determine the expression of different pro-angiogenic and growth factors in tumor extracts.

### Tumor measurement by caliper

Tumor volume was measured by digital caliper (Thermo Fisher Scientific, Rockford, IL, USA) on day 7, 14 and 21 after tumor implantation. The major longitudinal diameter (length) and the major transverse diameter (width) were determined. Tumor volume was calculated based on caliper measurements by the modified ellipsoidal formula [40]: Tumor volume  = ½ (length × width^2^)

### SPECT study

#### VEGF-c

Recombinant rat VEGF-c was purchased from Prospec (Rehovot, Israel). VEGF-C, known as Vascular Endothelial Growth Factor Related Protein (VRP), is a recently discovered member of VEGF growth factor family that is most closely related to VEGF-D. Similar to VEGF-D, VEGF-C has a VEGF homology domain spanning the middle third of the precursor molecule and long N- and C-terminal extensions. Recombinant rat VEGF-c, lacking the N- and C-terminal extensions and containing only the middle VEGF homology domain, forms primarily non-covalently linked dimers. This protein is a ligand for both VEGFR2 and VEGFR3.

#### Preparation of Hydrazine Nicotinamide (HYNIC)

Succinimidyl 6-hydrazinopyridine-3-carboxylate hydrochloride was synthesized and conjugated with rat VEGF-c as previously described [41]. The conjugate protein was purified with a Centricon C-3 diafilter with a 3,000 molecular weight cutoff. Then, the nicotinyl hydrazine conjugate of VEGF-c was radio labeled with 99m-Tc-pertechnetate in the presence of tricine and stannous chloride as reported by [42]–[43].

#### Image acquisition

An appropriate state of anesthesia was obtained using ketamine/xylazine (100/15 mg/kg). One hour after injection of 0.5 mCi Tc-99m-HYNIC-VEGF-c, SPECT images were obtained using a modified PRISM 3000 gamma camera dedicated to animal studies and fitted with multi-pinhole collimators (Bioscan, Washington DC, USA). The following image parameters were used: 360 degree rotation with 36 degree increments, 180 sec per projection, using 256×256 matrices with a field of view of 4×6 cm. Total SPECT image acquisition time was 10 minutes. After the SPECT analysis animals were euthanatized and the tumors was collected for further analysis. The projection images were reconstructed with HiSPECT software (Bioscan, Washington DC, USA).

#### Image analysis

Multi planar reconstruction and SPECT analysis were performed using ImageJ software (NIH, Bethesda MD, USA). The tumor center was identified using orthogonal views and all sections containing the tumor, either in axial or coronal views, were added. Total activity of Tc-99m-HYNIC-VEGF-c was determined by drawing irregular regions of interests (ROI) around the tumor and on the contralateral muscles to determine the activity in the contralateral side. The percentage of change in the total activity was calculated using the following formula:

(Mean activity in the total tumor volume/mean activity in the contralateral muscles)*100

### Protein extraction

Animals used for membrane antibody array analysis were euthanatized with 100mg/kg of pentobarbital administration (intravenous). The radioactive fluids were collected and contained in a shielded area to decay and the tumors were collected and snap frozen. Tissues from the tumors were mechanically pulverized over dry ice and total protein (80–150 mg of the frozen tissue powder per sample) was extracted using Ray Bio 2x Cell Lysis Buffer (RayBiotech, Norcross, GA, USA) according to the manufacturer's instructions. Protein concentration in recovered protein extracts was determined using Micro BCA Protein Assay Kit (Thermo Fisher Scientific, Rockford, IL, USA) using Bovine Serum Albumin as a standard.

### Membrane antibody array

Custom RayBio Human Cytokine Array kit (RayBiotech, Norcross, GA, USA) (Table 1) was used to analyze 20 protein expression in mammary tumors. All sample measurements were performed in duplicate, containing positive and negative controls.

**Table 1:** Custom designed protein arrays kit, which consist of 20 different cytokines/factors.

	A	B	C	D	E	F	G	H
**1**	Positive	Positive	Negative	Negative	Angiogenin	Angiostatin	Angiopoietin-1	Angiopoietin-2
**2**	Positive	Positive	Negative	Negative	Angiogenin	Angiostatin	Angiopoietin-1	Angiopoietin-2
**3**	G-CSF	PDGF-Ra	PDGF-AA	RANTES	bFGF	EGF	EGF R	IGF-I
**4**	G-CSF	PDGF-Ra	PDGF-AA	RANTES	bFGF	EGF	EGF R	IGF-I
**5**	MMP-9	SDF-1 α	Tie-1	Tie-2	VEGF-A	VEGF-C	VEGFR2	VEGFR3
**6**	MMP-9	SDF-1 α	Tie-1	Tie-2	VEGF-A	VEGF-C	VEGFR2	VEGFR3
**7**	Blank	Blank	Blank	Blank	Blank	Blank	Blank	Positive
**8**	Blank	Blank	Blank	Blank	Blank	Blank	Blank	Positive

G-CSF  =  colony stimulating factor; PDGF-Ra  =  platelet-derived growth factor receptor, alpha polypeptide; PDGF-AA  =  platelet-derived growth factor alpha polypeptide; bFGF  =  fibroblast growth factor (basic); EGF  =  epidermal growth factor; EGF R  =  epidermal growth factor receptor; IGF-I  =  insulin-like growth factor 1 (somatomedin C); MMP-9  =  matrix metallopeptidase 9; SDF-1 α  =  Stromal cell-derived factor 1 alpha; Tie-1  =  receptor tyrosine kinase of angiopoietin-1, tyrosine kinase with immunoglobulin-like and EGF-like domains 1; Tie-2  =  receptor tyrosine kinase of angiopoietin-1; VEGF-A  =  vascular endothelial growth factor A;VEGF-C  =  vascular endothelial growth factor C; VEGFR2  =  Vascular endothelial growth factor receptor 2; VEGFR3  =  Vascular endothelial growth factor receptor 3. All antibodies are prepared in duplicate.

Membranes were incubated in 8-well plates with 2 ml of RayBio 1X Blocking Buffer solution for 30 minutes. 500 ug of protein was added to each sample and the membranes were incubated overnight at 4°C. The solution was discarded and the membranes washed three times with 1X RayBio Wash Buffer I, and twice with 1X RayBio Wash Buffer II for 5 minutes each. BiotinConjugated Anti-Cytokines was added and samples were incubated overnight at 4°C. Membranes were washed with Wash Buffer I and II and incubated with 1000X HRP-Conjugated Streptavidin overnight at 4°C. After that, it was washed again with Wash Buffer I and II and incubated for 2 minutes with Detection Buffer. Membranes were exposed in a Multispectral In-Vivo Imaging System (Kodak™ Multispectral system, Carestream Health Inc., NY, USA).

The optical density of the expression of each protein was normalized to positive control and quantified using the ImageJ software (NIH, Bethesda, MD USA).

### Immunohistochemistry

Animals were euthanatized, perfused by intracardiac injection of 10 ml PBS followed by 10 ml 3% paraformaldehyde and tumors were removed and fixed in 3% paraformaldehyde containing 3% sucrose. Tumor sections were prepared for paraffin blocking and sectioning. Standard histochemical staining procedures were performed as recommended by the suppliers of primary antibodies. The following antibodies were used to delineate the expression of corresponding antigens: VEGFR2 (Millipore, Billerica, MA, USA), anti-VEGFR3 antibody (Millipore, Billerica, MA, USA), anti- von Willebrand Factor (vWF) (Millipore, Billerica, MA, USA) andanti-Ki67 (Millipore, Billerica, MA, USA).

### Evaluation of immunohistochemical staining

At least two sections from each tumor underwent staining for different markers and used for analysis. Multiple fields were examined from each slide, especially demarcated areas with brown staining. There were signs of necrosis in the central part of both melatonin and vehicle treated tumors.

For the analysis of immunohistochemistry slides, five areas were photographed at 40× magnifications (center, bottom, top, left and right regions) and saved in tiff format. VEGFR2 and VEGFR3 were analyzed based on the intensity of the staining by ImageJ software (NIH, Bethesda MD, USA).Each photograph was divided into four quadrants and 20 spots (small circular ROI) were randomly selected (avoiding the nucleus) in each quadrant, analyzing the intensity from 80 spots from each photographed area. The intensity was determined from a total of 400 spots marked on each slide. A negative control section of the corresponding staining was used for measuring background activity.

Evaluation of micro-vessel density (MVD) was detected by immunohistochemical staining with vWF. Each cell stained positive for vWF was considered to be a micro vessel. Five “hot spots” (area with highest vessel concentration) from each slide were identified and vWF positive areas were counted by two independent observers. The total histological area (mm^2^) was noted, and MVD was calculated as previously described [44].

Tumors were categorized in relation to cellular proliferation according to the staining of Ki-67.All Ki-67 positive cells were counted from each photographed area. The number of Ki-67 positive cells was normalized to the area of photomicrography.

### Statistical analysis

All data are expressed as mean ± standard error of mean (SEM). Comparison between melatonin and vehicle administration groups was done by Student's t-test or ANOVA followed by Bonferroni test with GraphPad Prism 4.0 software (La Jolla, CA, USA). Any p-value of 0.05 was considered significant.

## Results

### MTT assay

**Figure 1:**
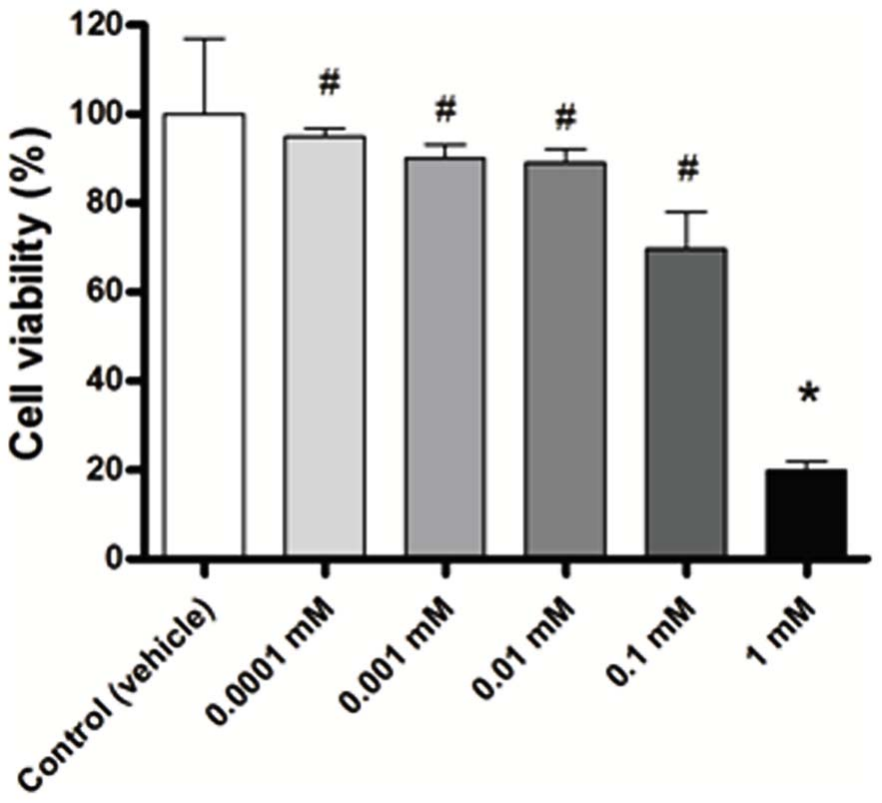
Inhibitory effect of melatonin on viability of the MDA-MB-231 cell line. The MDA-MB-231 cells were treated with five concentrations of melatonin for 24 h and cell viability was measured by MTT assay. Data are shown as mean ± S.D. *p<0.05, 1 mM of melatonin vs. Control; #p<0.05, 1 mM of melatonin vs other melatonin's concentrations.

We performed the MTT assay with different concentrations of melatonin to assess whether melatonin acts on the *in vitro* viability of MDA-MB-231 cells. As shown in Figure 1, treatment with 0.0001 mM to 0.1 mM of melatonin did not affect the cell viability after 24 hours (p>0.05). Only a pharmacological concentration of 1 mM of melatonin significantly decreased the cell viability compared to control cells (*p<0.05 vs control) and compared to all concentrations of melatonin evaluated (# p<0.05) (Figure 1).

### Tumor size

To evaluate whether melatonin treatment reduces the breast tumor growth *in vivo*, we implanted MDA-MB-231 cells in athymic nude mice and treated then with melatonin (40 mg/kg) or vehicle for 21 days.

None of the treated mice showed any loss of weight and lethargy during the treatment for 21 days. On the contrary, the treated animals showed excessive movement but no irritability or aggressive behavior.

The site of implantation of tumor cells (mammary gland or flank) did not alter the rate of tumor growth in the vehicle treated animals (p = 0.88) or in the melatonin-treated animals (p = 0.76), showing that there was no significant difference in tumor development and response to melatonin treatment between the two models used. Thus, the two models were grouped together into one group for each treatment (melatonin or vehicle) for subsequent analyses.

**Figure 2:**
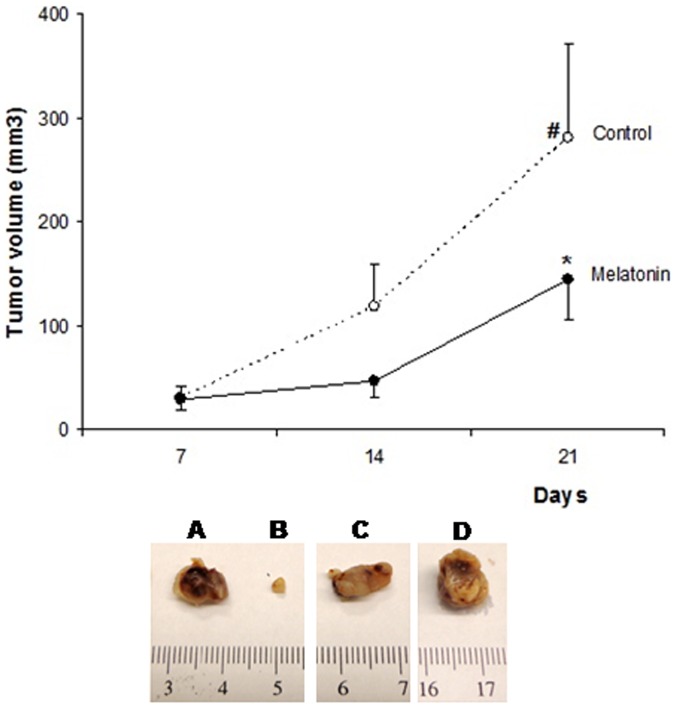
Antitumor effects of melatonin on mammary tumor growth. Melatonin reduced the tumor growth in breast cancer nude mice. Each point in the curves represents the mean ± SD (control n = 8; melatonin n = 5). The melatonin inhibited tumor growth, *p<0.05 vs Control. # Significant increase in tumor volume on control group at 14 and 21 after tumor implantation and initiation of treatment with vehicle (p<0.05). Detail: Representative samples of mammary tumors developed by MDA-MB-231 cells implantation on the right flank of mice. A, B. Melatonin treated mammary tumors, B. Mammary tumor which regressed with melatonin treatment. C, D. Vehicle treated mammary tumors.

Treated animals showed significantly smaller tumors after 21 days (p<0.05; **Figure 2**). The mean tumor volume of control and treated animals were 282.0±88.5 mm^3^ and 144.9±38.4 mm^3^, respectively. The mean tumor volume in control animals increased significantly from day 14 (118.9±40.2 mm^3^) today 21 (282.0±88.5 mm^3^), while this was not observed in the treated group (p<0.05, **Figure 2**). Furthermore, there was tumor regression in an animal treated with melatonin (Day 7 = 27.38 mm^3^; Day 14 = 8.79 mm^3^, Day 21 = 4.8 mm^3^) (**Figure 2B**). No similar pattern was seen in any of the control mice (**Figure 2C, 2D**).

### In vivo expression of VEGFRs

**Figure 3:**
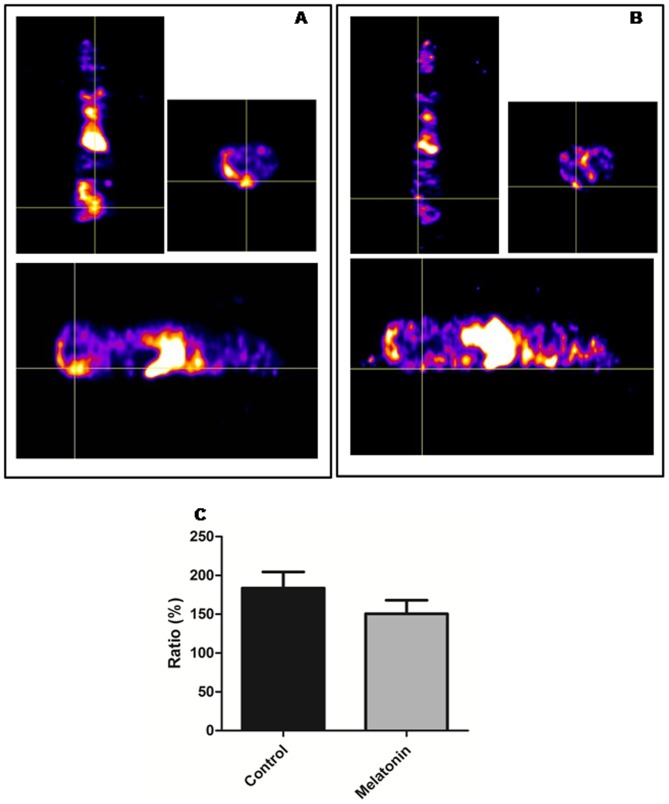
SPECT analysis of *in vivo* accumulation of Tc-99m-HYNIC-VEGF-c. VEGF-c (which targets both VEGFR2 and VEGFR3) was tagged with HYNIC chelators and then labeled with Tc-99m and injected intravenously in melatonin and vehicle treated mice. One hour after injection, SPECT images were obtained using dedicated animal scanner. Vehicle treated mice showed increased accumulation of Tc-99m-HYNIC-VEGF-c in the mammary tumor (A, Intersection of lines indicate the tumor, with a volume of 865.69 mm^3^ at the 21th day) compared to that of melatonin treated mammary tumors (B, Intersection of lines indicate the tumor, with a volume of 130.69 mm^3^ at the 21th day) C. Semi-quantitative analysis of total radioactivity normalized to contralateral muscles showing the intensity of radioactivity in the vehicle and melatonin treated animals.

To determine whether there were any changes in the expression of VEGFRs in the melatonin and vehicle administrated tumors, animals underwent SPECT scanning with Tc-99m tagged VEGF-c. Animals treated with melatonin showed lower activity of Tc-99-HYNIC-VEGF-c in the tumors compared to vehicle treated animals (**Figure 3A, 3B**).

Semi-quantitative analysis of total radioactivity normalized to contralateral muscles showed that the intensity of radioactivity in the control animals was 183.6±20.9%, while the intensity of radioactivity in animals treated with melatonin was 150.5±17.1%. Although there was difference in the radioactivity in the tumors between the groups, statistically significant difference was not achieved (p>0.05; **Figure 3C**).

### Membrane antibody array

**Figure 4:**
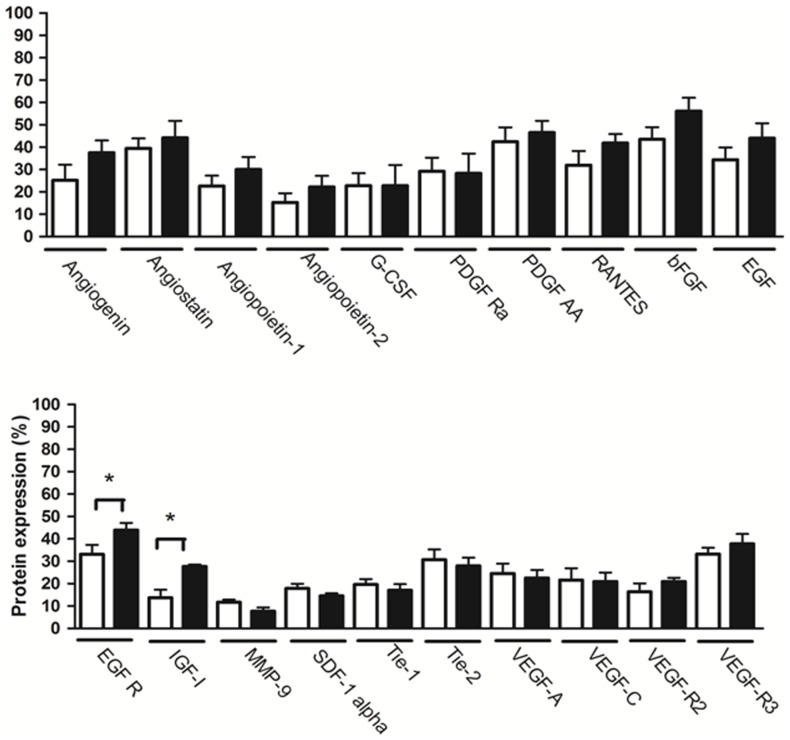
Comparison between proteins expression in mammary tumor in animals treated with melatonin or vehicle. White column  =  vehicle treatment; Black column  =  melatonin treatment. Data are shown as mean ± S.D. *p<0.05, vs. Control.

Semi-quantitative densitometry analysis of membranes showed that melatonin did not alter the pattern of expression of the majority of evaluated angiogenic proteins (p>0.05; **Figure 4**). However, melatonin increased the expression of epidermal growth factor receptor (EGFR) and insulin-like growth factor 1 (IGF-I) proteins (p<0.05; **Figure 4**).

### Immunohistochemistry

The expression of VEGFR2, VEGFR3, vWF and Ki-67 was evaluated for the tumors after 21 days of treatment with melatonin or vehicle by immunohistochemistry.

**Figure 5:**
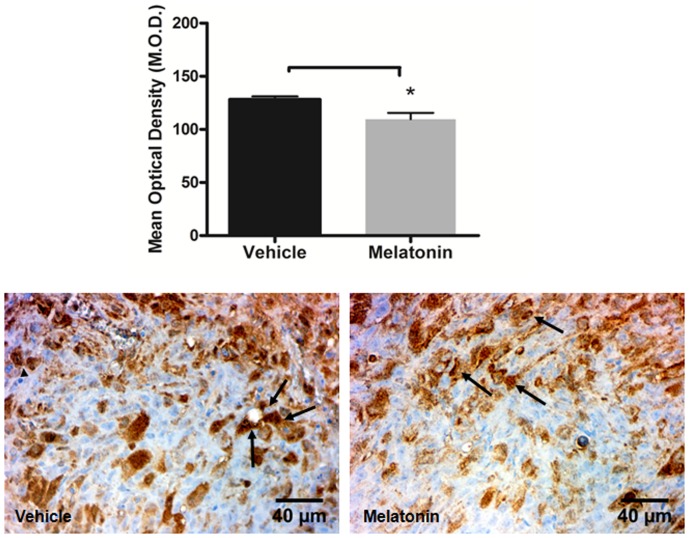
Immunohistochemistry staining with VEGFR2 (arrows) in vehicle treated and melatonin treated tumors. Images were taken with 40× magnification. A significant decrease was observed at the tumor in melatonin treated tumors compared to vehicle treated tumors (*p<0.05). Error bars: ± standard error.

**Figure 6:**
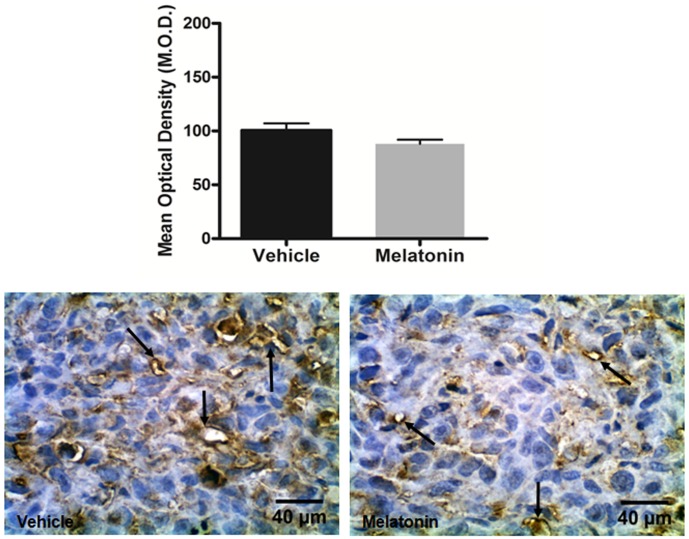
Immunohistochemistry staining with VEGFR3 (arrows) in vehicle treated and melatonin treated tumors. Images were taken with 40× magnification. Melatonin do not decreased significantly the expression of VEGFR3 (p>0.05). Error bars: ± standard error.

Lower expression of VEGFR2 was observed in the melatonin treated tumors compared to vehicle treated tumors (p<0.05; **Figure 5**). Furthermore, melatonin treated tumors exhibited lower expression of VEGFR3 compared with vehicle treated tumors, but statistically significant difference was not achieved (p>0.05; **Figure 6**).

**Figure 7:**
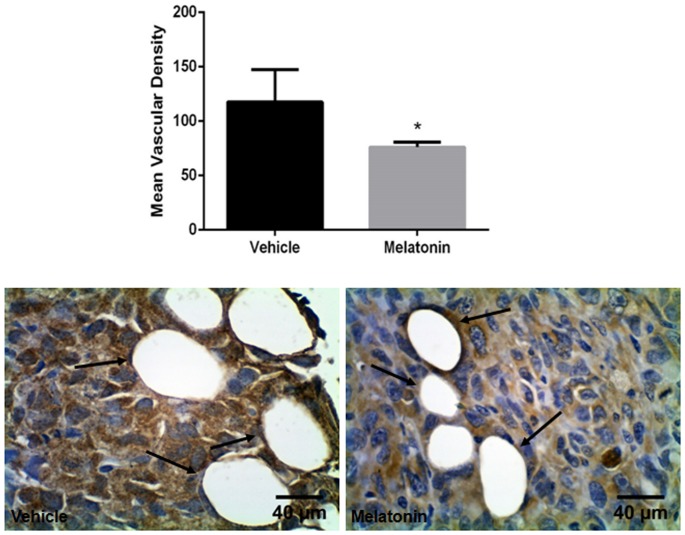
Immunohistochemistry staining with vWF in vehicle treated and melatonin treated tumors. Images were taken with 40× magnification. Quantitative estimation of micro-vessel density (MVD) by counting positive vessels (arrows) revealed a decrease in MVD after melatonin treatment compared to the vehicle treated tumor (*p<0.05). Error bars: ± standard error.

Tumor neovascularization was assessed by quantification of MVD in mammary tumors by vWF immunohistochemistry. Melatonin treatment resulted in a decrease of MVD compared to the vehicle treated tumors (p<0.05; **Figure 7**).

**Figure 8:**
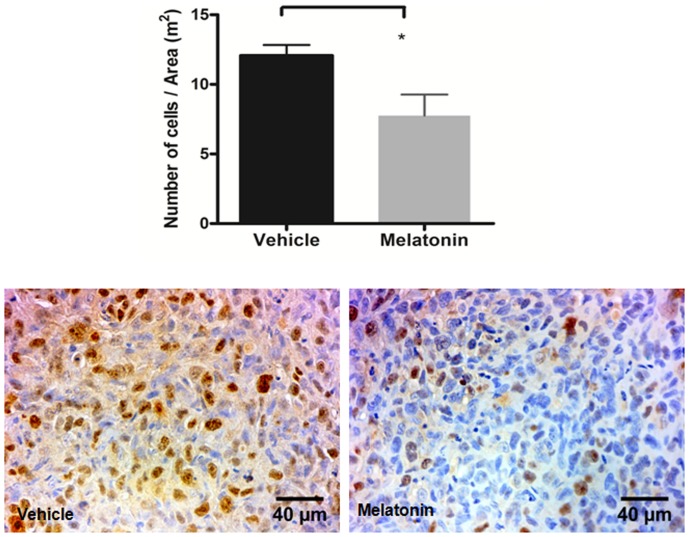
Immunohistochemistry staining with Ki-67 in vehicle treated and melatonin treated tumors. Images were taken with 40× magnification. There was a decreased cell proliferation in tumors treated with melatonin (*p<0.05). Error bars: ± standard error.

Numerous Ki-67 positive cells were observed in the vehicle treated tumors, whereas none to very few positive cells were observed in the melatonin treated tumors (p<0.05; **Figure 8**).

## Discussion

Results from this study showed that melatonin at pharmacologic concentration (1 mM) was able to reduce ER-negative breast cancer cell viability *in vitro*. The direct effects of melatonin on mammary cancer have been studied *in vitro*, basically using the ER-positive human breast cancer cell line (MCF-7) as a model [39]. In physiological concentrations (1 nM corresponding to peak nighttime and 10 pM corresponding to day time serum values in humans), melatonin suppresses the growth of ER-positive (MCF-7, T47D, ZR 75-1) and some ER-negative (MDA-MB-468) human breast cancer cell lines *in vitro* [45]. In addition, melatonin (10 uM – 1 mM) inhibited, to a varying extent, the proliferation of estrogen-responsive cell lines (MCF-7, T47D, ZR-75-1) more effectively than estrogen negative breast cancer cells such as BT-20, MDA-MB-231, MDA-MB-364, Hs587t, T47Dco, suggesting that the antiproliferative effects of melatonin are mediated through the estrogen-response pathway [39], [46].

However, agreeing with our results about ER-negative cells, Leman et al.[47] showed that melatonin at pharmacological level (1 mM) decreases the proliferation of MDA-MB-231 and MCF-7 cells significantly. Proliferation of MDA-MB-435 cells, which are highly metastatic and ER-negative, was not significantly affected by melatonin [47]. Jung et al. [48] showed that melatonin showed weak cytotoxicity only at pharmacologically high concentrations of 8 mM and 16 mM in MDA-MB-231 cells. Melatonin induced apoptosis and inhibited the proliferation via the inhibition of anti-apoptotic genes such as BCL-xL, Mcl-1, cyclin D1, cyclin E, p-STAT3, p-mTOR, and p-AKT at high concentration (12 mM) in ER-negative MDA-MB-231 cells [48].

Melatonin has been reported to bind and activate two distinct receptor types, membrane-bound G protein-coupled receptors, MT1 and MT2, and the nuclear orphan RZR/RORa receptors, members of the steroid/thyroid hormone receptor superfamily [49]. MT1 and MT2 are expressed in human cells in various organs and in neoplastic cells, including breast cancer cells [50].

Through the mediation of a subunit of G protein, MT1 receptors inhibit the activity of adenylcyclase, and thereby decrease the production of adenosine 3′, 5-cyclic monophosphate (cAMP). This relationship makes it possible to control the activity of selected protein kinases (PKC, PKA, MAPK) and to influence the levels of transcription factor phosphorylation, that is, cAMP response element-binding, as well as the expression of specific genes, which code proteins involved in the proliferation, angiogenesis, cell differentiation and migration processes [50].

Both MCF-7 and MDA-MB-231 human breast cancer cell lines express low levels of the MT1 receptor [46]; However, overexpression of the MT1 receptor enhanced the growth-inhibitory and gene-modulatory effects of melatonin in ER-positive but not ER-negative human breast cancer cells [49].

Recent studies have demonstrated higher levels of MT1A mRNA in the MCF-7 cell line compared to the MDA-MB-231 cell line [50], [51]. Jablonska et al. [50] showed that there is lower expression of MT1 receptors in breast cancer phenotype of triple negative (ER-, PR -, HER2 -) compared to ER-positive cases and the lower expression of MT1 is correlated with poor prognosis.

However, through activation of the MT1 receptor, melatonin can suppress the development of cancer via a broad spectrum of mechanisms with and without involvement of ER [45]. Oprea-Ilies et al. [52] showed that the MT1 positivity was associated with a lower stage and a smaller tumor size at time of diagnosis in triple-negative breast tumors patients.

Furthermore, melatonin can exert antitumoral properties by a set of complex mechanisms of action, not necessary involving the receptor pathway [26]. Melatonin may act directly, independently of its receptors or via them, making it difficult to understand the action of melatonin at the cellular level [26], [50].

Since melatonin decreased ER-negative breast cancer cell viability *in vitro*, we implanted cells in athymic nude mice. Our results showed that melatonin treatment (40 mg/kg) reduced the tumor growth with concomitant decreasing of cell proliferation (Ki-67).

There are no studies about melatonin treatment in ER-negative breast cancer *in vivo*, but investigators showed the effectiveness of melatonin in treating rats with breast cancer induced by a carcinogen DMBA [32]. In a study by Cos et al. [53] nude mice bearing ER-positive breast cancer (MCF-7) (inoculated directly into the inguinal mammary fat pad of the mouse) treated with melatonin also showed smaller tumor size compared to control animals. Rao et al. [54] showed that melatonin at 50 mg/kg and 200 mg/kg reduced the incidence of mammary cancer of TG.NK (c-neu) transgenic mice and reduced the number of tumors per mouse and tumor weights as compared with the control group. Although effective, the high-dose of 200 mg/kg caused significantly lower body weight with high mortality. On the contrary, in our study none of the treated mice showed any adverse effect, loss of weight and lethargy during the treatment with 40 mg/kg for 21 days. Liu et al. [55] performing a study with mice with gastric tumor (foregastric murine carcinoma cell line subcutaneously inoculated under the right axilla) showed that melatonin treatment in doses of 25 mg/kg, 50 mg/kg and 100 mg/kg reduced tumor volume when correlated with control mice.

It is accepted that melatonin exerts an anti-tumor effect and multiple mechanisms have been suggested for the biological effects of melatonin but are not yet fully established [56]. It has been shown that melatonin has direct anticancer mechanisms in several types of cancer, as pro-apoptotic, anti-proliferative, anti cell-differentiation and anti-angiogenic actions. Melatonin also has indirect anticancer mechanisms such as antioxidative effects and immune system regulation [26], [57]. Little is known about the anti-angiogenic effect of melatonin. Some authors have demonstrated that a pharmacological concentration of melatonin may inhibit angiogenesis directly or indirectly, by inhibiting the proliferation of vascular endothelial cells [33] and acting in the inhibition of pro-angiogenic factors [34]–[37].

In this study, we evaluated the action of melatonin on angiogenesis in ER-negative breast cancer *in vivo*. Our findings were able to show that melatonin treatment was effective in inhibiting ER-negative mammary tumor angiogenesis, indicated by the reduction of VEGFR2 expression and MVD. Despite the semi-quantitative analysis of Tc-99m-HYNIC-VEGF-c showing lower VEGFR2 expression in the melatonin treated tumors, a statistically significant difference was not achieved. The non-significant differences of Tc-99m-HYNIC-VEGF-c activity between control and treated tumors could be due to the reduction of tumor size in treated groups and the relative expression of VEGFR2 remained unchanged since our analysis used contralateral ROI identical to tumor size. However, a decrease of VEGFR2 in melatonin treated mice was confirmed by immunohistochemistry.

It is noteworthy that the efficacy of anti-angiogenic therapies can be evaluated by nuclear medicine imaging techniques using the tagging of radioactive isotopes to the specific proteins. Previously, Tc-99m was successfully tagged with different proteins and peptides using the new generation chelator, HYNIC (Hydrazine Nicotinamide) [43]. VEGF receptors have been used as imaging agents mediators of angiogenesis, since they are highly expressed in the vascular endothelium, and when injected into the bloodstream, are internalized by binding to VEGF, allowing the accumulation of contrast agents, such as Tc-99m [43], [58]. VEGFR2 is the principal receptor transmitting VEGF signals and is overexpressed in the tumor vasculature compared with normal vasculature [59]. One common mechanism for increased survival and growth of cancer cells is the deregulation of tyrosine kinase receptors like VEGFR2 [60].

In this study, mammary tumors showed necrosis in the central part, both in vehicle and melatonin treated animals, which can be a result of insufficient and defective vasculature in a highly proliferative tumor mass. Hypoxia in tumor tissues is a leading cause of angiogenesis [61]. Low O_2_ tension of a growing tumor allows stabilization of HIF-1α, leading to increased VEGF-A transcription, which binds to VEGFR2. Activation of intracellular signaling of VEGFR2 stimulates an angiogenic response, leading to cell proliferation, migration, permeability, survival and ultimately resulting in tumor growth [62].

There are no studies evaluating the expression of VEGFRs in response to treatment with melatonin. We also evaluated 20 angiogenic proteins in breast tumor tissue and showed that melatonin treatment did not alter the pattern of expression of the majority of the proteins evaluated. Previous reports have described a decrease in the production of VEGF protein induced by melatonin in human pancreatic carcinoma cells (PANC-1) and human alveolar adenocarcinoma cells (A549) [36], [56].

Melatonin suppresses tumor angiogenesis by inhibiting HIF-1α expression and stabilization in prostate cancer [37] and HCT116 colon cancer cells under hypoxic condition *in vitro* [5]. We evaluated the expression of HIF-1α in tumor tissue of animals treated with melatonin or vehicle, but there was no statistically significant difference (p>0.05, data not shown).

Melatonin suppressed the VEGF gene expression through the inhibition of the accumulation of HIF-1a only under a hypoxia environment and had no obvious effect under normoxic conditions [63] and in physiological concentrations [56].

Regarding breast cancer, Alvarez-García et al. [64] performed an *in vitro* study and showed that melatonin may play a role in the paracrine interactions between malignant epithelial cells and proximal endothelial cells through a downregulatory action on VEGF expression in MCF-7, which decreases the levels of VEGF around endothelial cells. Lower levels of VEGF could be important in reducing the number of estrogen-producing cells proximal to malignant cells.

Kim et al. [61] showed that melatonin treatment suppressed tumor angiogenesis and diminished the formation of the capillaries inside the tumor, reducing the tumor growth in mice bearing renal adenocarcinoma (RENCA). In addition, it has also been reported in rats injected with melatonin while exposed to hypoxia, that serum VEGF levels decreased, which suggests that melatonin can suppress tumor cellular hypoxic adaptation by inhibiting angiogenesis [35]. It is notable that a decrease in the expression of HIF-1 and its target gene VEGF can happen by antioxidant action of melatonin, removing intracellular reactive oxygen species.

These studies indicate that the regulation of VEGF by melatonin is highly complex. Many factors are involved in the regulation of VEGF synthesis, but the relation between melatonin and VEGF has been unclear until now. Perhaps, melatonin receptors (membrane and nuclear), and its well-known immunomodulatory role and other additional transcriptional factors are involved in the actions of melatonin [56].

In addition, semi-quantitative densitometry analysis of membranes showed that melatonin treatment increased the expression of EGFR and IGF-I proteins in ER-negative breast cancer. Margheri et al. [65] demonstrated that the anticancer effects of melatonin on breast cancer cell line MCF-7 were enhanced by its combination with retinoic acid and somastatin. The combined treatment resulted in a decrease in cell viability, which was concomitant with inhibition of a signalling pathway mediated by EGF. The oncostatic action of melatonin in ER-positive breast cancer cells MCF-7, probably occurs via its binding to the MT1 receptor and the alteration of key kinase pathways, for example IGF-I-PI3K/Akt, which were shown to be involved in the estrogen response pathway of the tumor cell [50].

IGF-I is a growth factor that stimulates the growth of breast cancer. Rao et al. [54] showed that melatonin (50 mg/kg, 100 mg/kg, 200 mg/kg) did not cause significant changes in serum IGF-1 levels in mammary cancer of TG.NK (c-neu) mice. Lissoni et al. [66] developed a clinical phase II, in which it was demonstrated that, in patients with metastatic breast cancer, tamoxifen therapy in conjunction with melatonin resulted in the decrease of serum levels of IGF-I.

In this study we proved that melatonin reduces tumor angiogenesis in an ER-negative breast cancer model, but the detailed mechanism of how melatonin acts on angiogenesis in various cancers should be investigated in depth in further studies. Taken together, our results showed that melatonin inhibits tumor growth, cell proliferation and blocks tumor angiogenesis in breast cancer and the anti-tumor action of melatonin. Furthermore, melatonin treatment has not caused systemic toxicity to achieve the therapeutic activities, suggesting it as a potential therapeutic agent to breast cancer.
